# Comparison of Intraoperative Fentanyl Usage and Waste After Transition from 100-μg Vials to 50-μg Preloaded Syringes: A Single-Center Retrospective Study

**DOI:** 10.11648/j.ijacm.20241202.18

**Published:** 2024-11-22

**Authors:** Huang Huang, Emily Lai, Shreyas Bhavsar, Brian Miller, Jovelle Chung, Bradly Phillips, Lei Feng, Jose Miguel Soliz, Jessica Brown

**Affiliations:** 1 Department of Anesthesiology and Perioperative Medicine, The University of Texas MD Anderson Cancer Center, Houston, USA; 2 Department of Pharmacy, The University of Texas MD Anderson Cancer Center, Houston, USA; 3 Department of Biostatistics, The University of Texas MD Anderson Cancer Center, Houston, USA

**Keywords:** Opioids, Waste, Medication Vial Size, Preloaded Syringe, Analgesia

## Abstract

**Background:**

The rapidly acting opioid fentanyl commonly used in the perioperative setting, has traditionally been packaged in 100 or 250-μg vials. In September 2021, our institution implemented a change from fentanyl 100-μg vials to 50-μg preloaded syringes in our operating rooms. The objective of this study was aimed at assessing the association of the fentanyl product change on reducing medication waste and the amount of fentanyl administered during surgery.

**Methods:**

This single-center, retrospective study entailed a review of anesthesia records from September 2020 to September 2022 of adult patients who underwent general anesthesia and received fentanyl for surgery at The University of Texas MD Anderson Cancer Center. The data set was divided into a control period (CP) using 100-μg vials and a post transition period (PT) using 50-μg preloaded syringes. The primary outcome measures were the average amounts of fentanyl used and wasted per case. Secondary outcome measures consisted of intraoperative analgesic use as well as postoperative pain scores.

**Results:**

Among the 33,405 cases included in this study, the mean amount of fentanyl used per surgical case was higher in the CP group than in the PT group (133μg vs. 102μg; p<0.001). Additionally, fentanyl waste occurred in a higher percentage of cases in the CP group than in the PT group (13.9% vs. 2.9%; p<0.001). We did not observe a significant difference in post-anesthesia care unit pain scores between the CP and PT groups.

**Conclusion:**

Transitioning to preloaded fentanyl syringes decreased medication waste and overuse of opioids intraoperatively. Simultaneously, the transition did not adversely affect patient analgesia in the post-anesthesia care unit.

## 1. Introduction

Adverse clinical events leading to over administration of injectable medications have resulted in deleterious errors leading to increased hospitalization and health care costs, estimates of the latter ranging from $2.7 billion to $5.1 per year. [1, 2] Opioid use in particular has been a major focus and contributor to adverse patient outcomes and increased health care costs. [3, 4] Perioperative opioid use can cause a multitude of negative side effects, including postoperative nausea and vomiting, respiratory depression, and decreased bowel motility, with resultant increases in readmission of surgical patients and negative hospital patient experience. [5] In the clinical context, proper disposal and reconciliation of unused perioperative opioids have been linked with increases in time and cost for providers in reporting and tracking the waste of controlled substances. [6] Prevention of drug diversion is another important objective in addressing the opioid epidemic. [7] Furthermore, drug waste poses a serious risk to the environment, with harmful ecological implications and a burden to soaring health care costs. [8]

One of the most commonly used intravenous medications in the perioperative setting is fentanyl, a potent synthetic opioid that is valued in the field of anesthesiology for its powerful and rapid-acting analgesic properties. Traditionally, this medication has been supplied in 250 or 100-μg glass vials, enabling anesthesiologists to draw up the necessary dose for each patient prior to administration. The remainder of the vial is discarded to adhere to sterility guidelines, which prevents the possibility of cross-contamination or infection. Although clinically safe, this practice has generated a considerable amount of waste, particularly in cases where the patient's required dose is significantly lower than the total vial amount. In addition, the process of disposing of fentanyl involves witness documentation, dedicated medication waste bins, and a reconciliation process required by pharmacy.

In September 2021, our institution fully implemented a change in the administration of fentanyl, transitioning from the conventional 100-μg vial to a 50-μg preloaded, sealed syringe format. This modification was driven by an aim to increase the precision of dosing and reduce the potential waste of fentanyl, which not only has cost implications but also addresses environmental concerns and aligns with sustainable health care practices. In this retrospective study, we compared the fentanyl usage and waste data along with pain scores before and after the transition to 50-μg fentanyl syringes. Our hypothesis was that this change, we could decrease waste at our institution. In doing so, we also hoped to gain insight into the effectiveness of this intervention and guide future initiatives aimed at improving efficiency, reducing waste, and providing the highest standard of patient care.

## 2. Methods

After obtaining Institutional Review Board approval from The University of Texas MD Anderson Cancer Center (protocol #2023-0780), a retrospective review of all operating room surgical cases from September 2020 to September 2022 was performed. All adult patients (age ≥18 years) who underwent an operating room procedure and received intravenous fentanyl under general anesthesia were included. Cases documented as monitored anesthetic care and emergency cases were excluded. The collected data were divided into two groups for comparison: the control period (CP), in which 100-μg vials of fentanyl were used, and the post transition period (PT), in which 50-μg syringes preloaded with fentanyl were used. The primary outcome measures the average amount of fentanyl used per case and the amount of fentanyl wasted per case as documented in the anesthesia electronic record and pharmacy waste report. Secondary outcome measures consisted of intraoperative analgesic use and postoperative pain scores. Postoperative pain scores were obtained from each patient’s electronic medical record.

## 3. Statistical Analysis

Statistical analyses were performed using SAS software (SAS Institute). Descriptive statistics were summarized. The Student *t*-test or Mann-Whitney *U* test was used to compare continuous variables, whereas a chi-square test or the Fisher exact test was used to compare categorical variables. P-values less than 0.05 were considered significant. For comparison of postoperative pain scores, a visual analog scale score difference greater than 1.6 was considered clinically significant. [9]

## 4. Results

We included a total of 33,405 cases in this study. The number of cases in the CP group was 16,603, whereas that in the PT group was 16,802. The patients’ baseline characteristics are presented in Table 1. Patients in the PT group were older than the CP group (59.28 vs. 58.90 years; p=0.007), and more of them had an American Society of Anesthesiologist physical status score of at least 3 (88.3% vs. 87.5%; p=0.037). The use of regional anesthesia was not significantly different in the two groups (p=0.528). The differences in the types of surgery are also described in Table 1.

**Figure 1.**
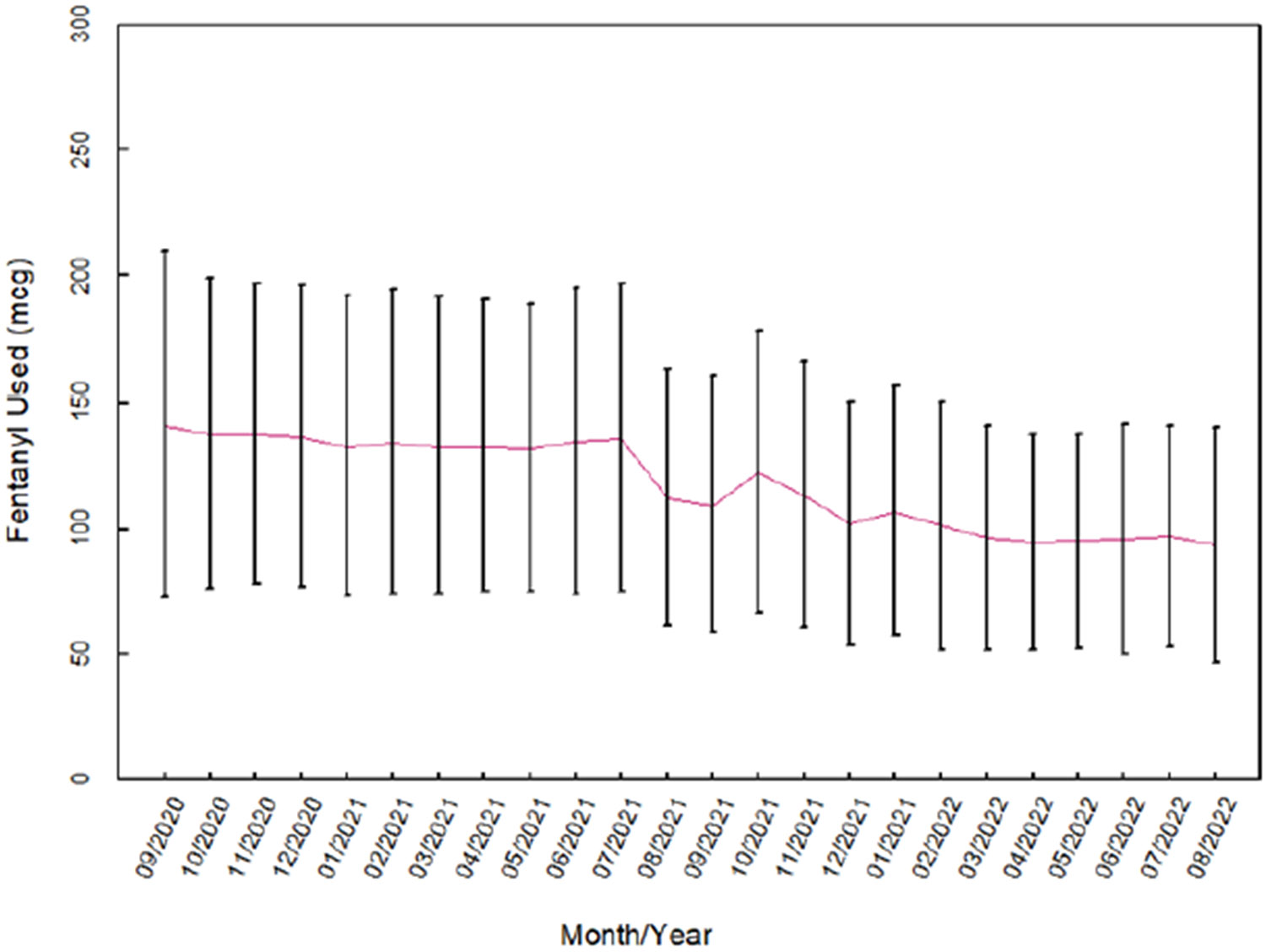
Average fentanyl use per surgical case by month.

**Figure 2.**
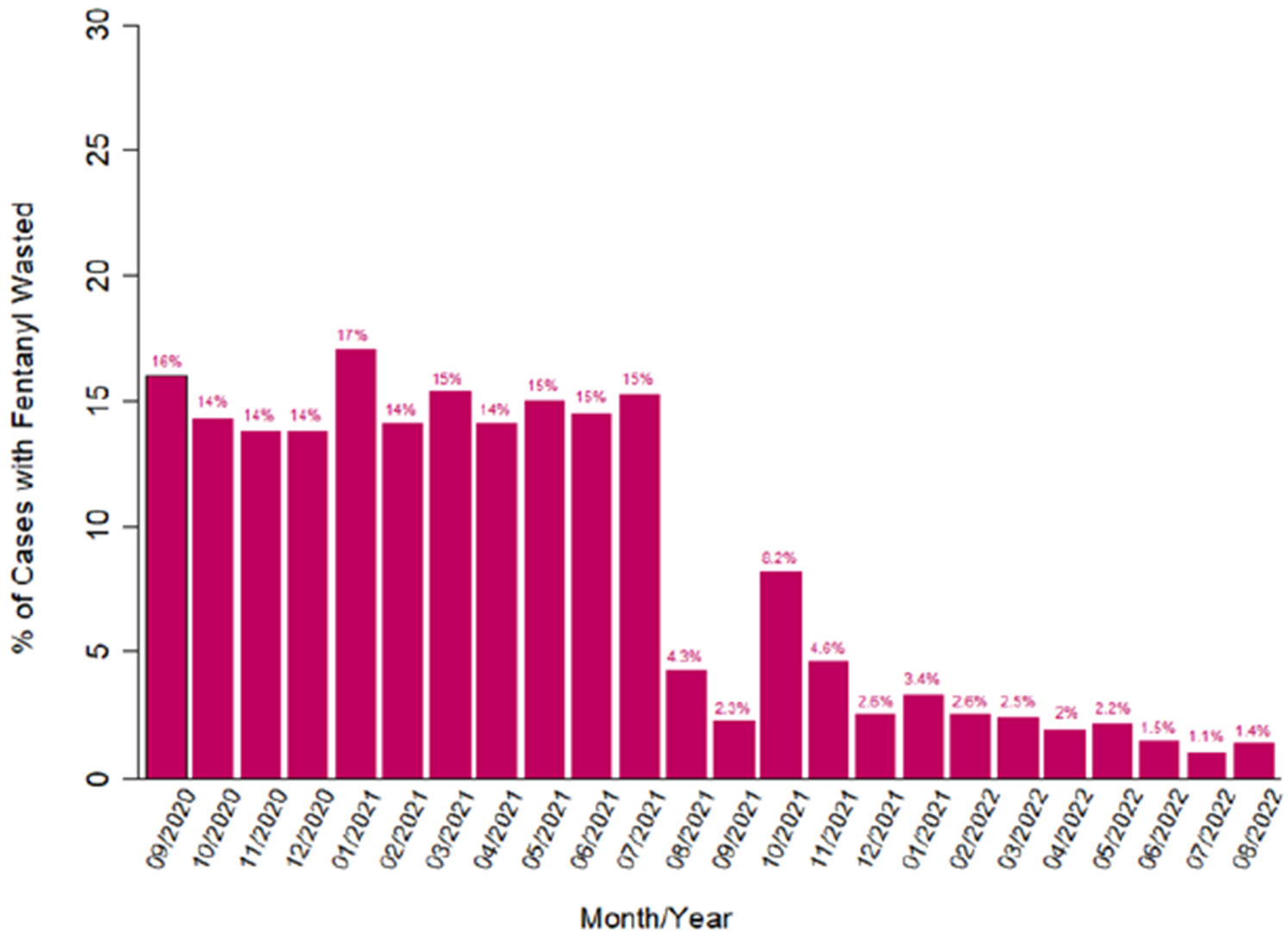
Percentages of surgical cases with fentanyl waste by month.

The mean amount of fentanyl used per surgical case was higher in the CP group than in the PT group (133 μg vs. 102 μg; p<0.001). Figure 1 shows the trend in fentanyl use per case over time during the entire study period. The percentage of cases with any amount of fentanyl waste was higher in the CP group than in the PT group (13.9% vs. 2.9%; p<0.001). Likewise, the percentage of cases in which a full fentanyl package waste occurred was higher in the CP group than in the PT group (2.1% vs. 0.5%; p<0.001). This trend over the study period is shown in Figure 2.

The use of opioids other than fentanyl and of nonnarcotic medications prior to or during surgery in the study patients is summarized in Table 2. The PT group had a significantly higher percentage of patients given hydromorphone (48.0% vs. 46.5%; p=0.008). However, the mean amount of hydromorphone given per case was not significantly different (p=0.076). In addition, the PT group had a significantly lower percentage of patients given several nonnarcotic medications such as acetaminophen, tramadol, and ketorolac. When comparing the difference in the post-anesthesia care unit (PACU) pain scores, the initial pain scores groups were not statistically or clinically significant between the CP and PT groups. In addition, the differences between the CP and PT groups in the median (2.14 vs. 2.05; p=0.006), maximum (4.27 vs. 4.46; p<0.001), and last (1.81 vs. 1.68; p<0.001) PACU pain scores were statistically but not clinically significant (Table 3).

## 5. Discussion

The aim of the transition from conventional fentanyl vials to preloaded syringes at our institution was to address the challenges of medication waste and increase the precision of dosing in the intraoperative setting. Our study demonstrated a significant reduction in fentanyl waste, with notably less overall waste and full package waste in the PT group than in the CP group. Managing opioid waste is an important factor to consider in the ongoing goal of mitigating the national opioid epidemic and drug diversion. It is noted that opioids have high wastages rates (e.g., 26.3-57.5% for morphine). As such, decreasing the syringe or vial size and hence opioid waste can deter drug diversion. [10] Important points of vulnerability in diversion of medications include drug preparation, administration, documentation, and wasting. Specific strategies that have used to minimize the possibility of diversion thru the wasting process include changing the medication for other substitutes, witness wasting procedures, and waste reconciliation audits. Regarding healthcare costs, opioid waste has been associated with increased costs owing to both pharmaceutical product waste and the time needed for personnel to report and reconcile it. [11, 12, 13]

Studies have demonstrated that reducing the aliquot size of drugs reduces wastage. For instance, a study at a tertiary care medical center demonstrated that eliminating 50-mL and 100-mL propofol vials from the formulary and replacing them with 20-mL vials reduced propofol waste by 45%. [14] Additionally, a retrospective study demonstrated that larger fentanyl vial sizes were associated with more intraoperative fentanyl administration. [15] Decreasing fentanyl aliquot sizes and prepackaging fentanyl in syringes rather than glass vials seem to have caused a behavioral shift among providers.

A preloaded syringe offers several benefits over glass vials, including convenience, time savings, reduced risk of medication errors, and improved safety owing to limitation of the need to puncture vials, thus reducing the risk of needle stick injuries. Furthermore, this transition could potentially reduce the overuse of fentanyl by allowing anesthesiologists to administer the smallest effective dose more accurately, which in turn may decrease postoperative recovery time, improve patient satisfaction, and contribute to a reduction in the opioid burden overall.

The limitations of our study include the retrospective design and the fact that the data set was extracted from a single institution. The retrospective nature of this study may have led to confounding variables that were not accounted for. Whereas the large data set allowed for minimizing of co-founding variables, it also resulted in small differences in demographics and surgical data becoming significant. This is noted in statistically significant age differences (58.9 years in the CP group and 59.2 in the PT group), and surgery types differences between the study groups. Additionally, some patients may have received other types of intraoperative opioids in addition to fentanyl, such as hydromorphone, remifentanil, and sufentanil, which may have affected the initial PACU pain score. Also, we did not find significantly larger amounts of opioid or nonopioid medications given per case in the PT group than in the CP group. However, the amounts of ketamine, celecoxib, and tramadol per case were markedly lower in the PT group. Moreover, the amounts and types of analgesics given in the PACU were not captured by our data extraction. Examining this information could have provided additional insight regarding the possible need for supplemental medications in recovery. Nevertheless, the comparable PACU pain scores in the CP and PT groups indicate that the transition to fentanyl-preloaded syringes did not adversely affect patients’ pain management.

## 6. Conclusion

In conclusion, the transition from 100-μg vials of fentanyl to preloaded 50-μg syringes was associated with a decrease in the amount of intraoperative fentanyl waste and administration without significantly affecting postoperative pain scores. Future directions for research could include investigating the role of reduced opioid waste in reducing the cost of wasted medications to the health care system. Moreover, future studies could analyze whether indirect costs could be decreased by reducing time spent by pharmacy staff in resolving controlled substance discrepancies and the decreased need for personnel credentialed in witnessing fentanyl waste.

## Figures and Tables

**Table 1.** Baseline characteristics and primary outcomes for the study patients.

Variable	CP (n = 16,603)	PT (n = 16,802)	p-value
Mean (± SD) age, years	58.90 ± 13.98	59.28 ± 13.99	0.007
Mean (± SD) BMI, kg/m^2^	29.04 ± 6.56	29.07 ± 6.57	0.809
Sex, n (%)			0.258
Male	9859 (59.4)	9875 (58.8)	
Female	6744 (40.6)	6927 (41.2)	
ASA, n (%)			
1 or 2	2069 (12.5)	1969 (11.7)	0.037
3, 4, or 5	14,534 (87.5)	14,833 (88.3)	
Mean (± SD) length of surgery, minutes	238.25 ± 161.82	240.42 ± 165.89	0.613
Surgery type, n (%)			
Breast	2557 (15.4)	2736 (16.3)	
Colorectal	766 (4.6)	861 (5.1)	
Gynecologic	1517 (9.1)	1478 (8.8)	
Head and neck	2157 (13.0)	2133 (12.7)	
Neurosurgery	820 (4.9)	788 (4.7)	0.016
Orthopedics	595 (3.6)	635 (3.8)	
Plastic surgery	1990 (12.0)	1862 (11.1)	
Surgical oncology	2601 (15.7)	2672 (15.9)	
Thoracic surgery	796 (4.8)	756 (4.5)	
Urology	2804 (16.9)	2881 (17.1)	
Regional anesthesia use, n (%)	852 (5.1)	888 (5.3)	0.528
Mean (± SD) fentanyl use per case, μg	133.14 ± 60.02	102.47 ± 48.72	<0.001
Cases with any fentanyl waste, n (%)	2309 (13.9)	492 (2.9)	<0.001
Fentanyl full package waste, n (%)	342 (2.1)	87 (0.5)	<0.001

*ASA*, American Society of Anesthesiologist physical status score; *BMI*, body mass index

**Table 2.** Opioid and non-narcotic medication use by the study patients.

Medication	CP (n = 16,603)	PT (n = 16,802)	p-value
Hydromorphone			
Number of patients (%)	7726 (46.5)	8062 (48.0)	0.008
Mean (± SD) dose, mg	0.88 ± 0.53	0.86 ± 0.51	0.076
Morphine			
Number of patients (%)	8 (0)	3 (0)	0.144
Mean (± SD) dose, mg	2.94 ± 1.85	4.00 ± 2.00	0.519
Remifentanil			
Number of patients (%)	432 (2.6)	409 (2.4)	0.328
Mean (± SD) dose, μg	0.91 ± 0.63	0.86 ± 0.57	0.302
Ketamine			
Number of patients (%)	2015 (12.1)	2002 (11.9)	0.534
Mean (± SD) dose, mg	76.34 ± 42.28	71.53 ± 39.05	<0.001
Acetaminophen			
Number of patients (%)	12,372 (74.5)	12,138 (72.2)	<0.001
Mean (± SD) dose, mg	1068.00 ± 298.00	1072.00 ± 306.00	0.632
Celecoxib			
Number of patients (%)	3889 (23.4)	4180 (24.9)	0.002
Mean (± SD) dose, mg	275.00 ± 103.00	224.00 ± 73.00	<0.001
Tramadol			
Number of patients (%)	2603 (15.7)	2325 (13.8)	<0.001
Mean (± SD) dose, mg	189.00 ± 158.00	158.00 ± 73.00	<0.001
Ketorolac			
Number of patients (%)	539 (3.2)	460 (2.7)	0.006
Mean (± SD) dose, mg	25.06 ± 7.25	25.13 ± 7.65	0.952

**Table 3.** PACU pain scores for the study patients.

Variable	CP	PT	p-value
Initial PACU pain score			
Mean (± SD)	1.60 ± 2.82	1.57 ± 2.80	0.893
Median (IQR)	0 (0-2)	0 (0-3)	
Median PACU pain score			
Mean (± SD)	2.14 ± 2.24	2.05 ± 2.12	0.006
Median (IQR)	2 (0-3.5)	2 (0-3)	
Maximum PACU pain score			
Mean (± SD)	4.27 ± 3.20	4.46 ± 3.22	<0.001
Median (IQR)	5 (0-7)	5 (0-7)	
Last PACU pain score			
Mean (± SD)	1.81 ± 1.92	1.68 ± 1.76	<0.001
Median (IQR)	2 (0-3)	2 (0-3)	

CP: control period. PT: post transition period

## Abbreviations

CP: Control Period
PT: Post Transition Period
ASA: American Society of Anesthesiologist Physical Status Score
BMI: Body Mass Index
